# Role of Active Site and CO_2_‐Interacting Surface Species in Dry Reforming of Methane over Strontium Promoted Ni Catalyst Supported by Lanthanum‐Zirconia

**DOI:** 10.1002/open.202400151

**Published:** 2024-11-06

**Authors:** Kenit Acharya, Ahmed S. Al‐Fatesh, Anis H. Fakeeha, Ahmed E. Abasaeed, Othman Alothman, Hammad Ahmad Jan, Naif Alarifi, Jehad K. Abu‐Dahrieh, Rawesh Kumar

**Affiliations:** ^1^ Department of Chemistry Indus University Ahmedabad Gujarat 382115 India; ^2^ Chemical Engineering Department College of Engineering King Saud University P.O. Box 800 Riyadh 11421 Kingdom of Saudi Arabia; ^3^ Department of Botany University of Buner Swari Pakistan; ^4^ Institute of Refining and Petrochemicals Technologies King Abdulaziz City for Science and Technology (KACST) P.O. Box 6086 Riyadh 11442 Kingdom of Saudi Arabia; ^5^ School of Chemistry and Chemical Engineering Queen's University Belfast BT9 5AG UK

**Keywords:** Sr promoter, Dry reforming of methane, Lathana-zirconia, Active site Ni, Ionic CO_3_^2−^ species

## Abstract

In the context of global warming, the dry reforming of methane (DRM) has gained significant attention due to its ability to simultaneously deplete two greenhouse gases, i. e. CH_4_ and CO_2_, and generate syngas. Herein, strontium‐promoted Lanthanum‐zirconia supported Ni catalysts are investigated for DRM and characterized by X‐ray diffraction, surface area and porosity, FTIR‐RAMAN spectroscopy, and temperature‐programmed experiments. The Ni/LaZr catalyst contains formate and oxycarbonate‐like CO_2_‐interacting species, while strontium‐promoted catalysts have additional ionic CO_3_^2−^ species. The current catalyst system of 2 % strontium‐promoted Ni/LaZr has active sites derived from three types of NiO: easily reducible, moderately interacted, and strongly interacted. During the DRM reaction over the current system, CO_2_ is a better oxidant than O_2_ for removing carbon deposits. Additionally, the catalysts attain higher reducibility under oxidizing gas (CO_2_) and reducing gas (H_2_) during the DRM reaction. For optimal hydrogen yield of approximately 60 % within 420 minutes of operation over Ni2Sr/LaZr catalyst, a balance between the population of active site Ni and CO_2_‐interacting surface species is necessary.

## Graphical abstract

The Role of active and CO_2_‐interacting species for dry reforming of methane over strontium‐promoted Ni/LaZr is investigated. The high activity over 2 wt % strontium‐promoted Ni/LaZr catalyst is due to the additional presence of CO_3_^2−^ species (along with other CO_2_‐interacting species) and strongly interacted NiO species (along with moderately and weakly interacted NiO species). However, the excessive presence of ionic carbonate species (over >2 wt % Sr promoted Ni/LaZr catalyst Ni) shows inferior activity towards DRM.

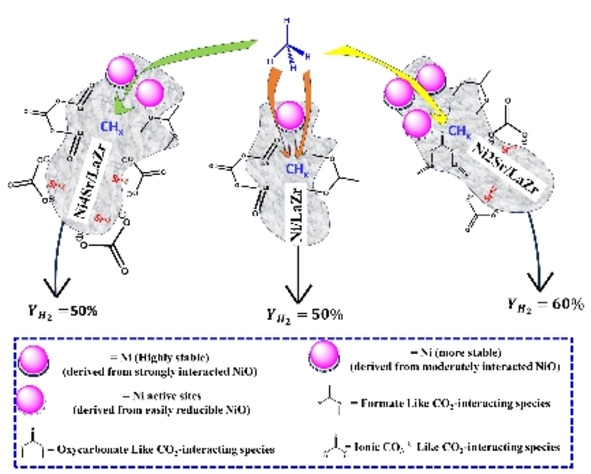

## Introduction

The concentration of greenhouse gases has surpassed critical levels, endangering the entire planet by global warming. It brought drastic seasonal changes and a rise in the sea, which seriously affected the ecological life cycles and agricultural outcomes.^[1]^ In the meantime, the catalytic conversion of dry reforming of methane gives hope for the potential transformation of greenhouse gases such as CH_4_ and CO_2_ into H_2_‐rich syngas (CH_4_+CO_2_→2CO+2H_2_; ΔH=247 kJ/mol). In this way, the concentration of both CH_4_ and CO_2_ can be depleted, and the clean energy goals with H_2_ can be achieved together.^[2]^

The DRM is an endothermic reaction and needs 600–850 °C reaction temperature. At this temperature range, more than 8 active metals, 12 supports, and 35 promotors are identified for dry reforming of methane reaction.^[3]^ However, low‐cost Ni as an active metal has gained the widest attention from the research community. Even thermally stable Ni foam could carry a CH_4_ decomposition reaction.^[4]^ Alumina, silica, and zirconia are the most investigated catalyst supports for Ni catalysis in this reaction. To influence the basicity‐reducibility‐stability of the catalyst without masking the catalytic active sites, an optimum amount of promoter is added. Group II metal oxide is used frequently for in‐building basicity in a material. Larger Sr^2+^ is also known for stabilising larger anions’ CO_3_^2−^, and SrO has an inherent basicity for interaction with CO_2_.[^5^, ^6^] However, alumina‐supported SrO can carry more CO_2_ than unsupported SrO.^[7]^ The promotion addition of Sr over Rh−Mn/Al_2_O_3_ induces higher reducibility, Sr over Fe/SiO_2_‐Al_2_O_3_ brought enhanced CH_4_‐decomposition, and Sr over Ni/Al_2_O_3_ nurtured strong metal support interaction and basicity.[^8^, ^9^, ^10^] Adding 5 wt.% Sr to Ni/La_2_O_3_ induced CO_2_ adsorption due to surface oxygen species, leading to C−H dissociation.^[11]^ It resulted in a 40 % H_2_ yield. The zirconia‐based support had gained increasing interest due to lattice oxygen endowing capacity at high temperatures. The interaction between zirconia and strontium was impressive as it resisted the sintering of Sr−O during CO_2_ sorption at high temperatures.^[12]^ The sorption behaviour of hydrous zirconium dioxide for strontium has been previously reported.^[13]^ A 64.5 % H_2_ yield was achieved using a higher Ni/Sr ratio (0.33) over yttria‐zirconia at 600 °C for 10 hours.^[14]^ Using lanthana along with major zirconia may have additional benefits like stabilising the zirconia phase, providing CO_2_ adsorption on additional La^+3^ sites and potentially decomposition CH_4_ at wide Ni‐LaZr interface.^[15]^

Although ZrO_2_ has oxygen‐endowing capacity, but instability against high temperatures is a major limitation of using ZrO_2_ as support. It has been found that the addition of La and Sr to Ni/ZrO_2_ enhances the interaction with CO_2_, improves reducibility, promotes CH_4_ decomposition, and increases catalytic stability. To create the support material, 9 wt.% La_2_O_3_ and 91 wt.% ZrO_2_ were mixed, resulting in a material referred to as LaZr. Next, 5 wt.% Ni was impregnated onto the LaZr support. Finally, a promotion addition of 1–4 wt.% Sr over 5Ni/LaZr was carried out using the impregnation method. Catalysts are characterized by X‐ray diffraction, surface area, and porosity techniques, Fourier transform Infrared spectroscopy, Raman spectroscopy, and temperature‐programmed oxidation‐reduction‐desorption techniques. The fine characterization data correlation with catalytic activity presents the applicability of using a strontium‐based promoter over lanthanum‐zirconia supported Ni catalyst for dry reforming of methane.

## Experimental Methods

### Materials

Ni(NO_3_)_2_.6H_2_O (98 %, Alfa Aesar), Sr(NO_3_)_2_ 6H_2_O (98 %, Alfa Aesar), lanthanum oxide (La_2_O_3_‐ Chengdu Beyond Chemical CO. LTD, China), zirconium oxide (Zr_2_O_3_) (99.9 %, Alfa Aesar), and deionized water.

### Catalyst Preparation

9 wt.% Lanthana and 91 wt.% zirconia support is prepared by mechanically mixing the respective metal oxides. A solution containing nickel nitrate (equivalent to 5 wt.% Ni) and another solution containing 1–4 wt.% of strontium nitrate is added to the support material using the impregnation method. The mixture is then stirred at 80 °C and dried overnight at 120 °C. Finally, the dried product is calcined at 600 °C for 3 hours. The resulting catalysts are called Strontium‐promoted Lanthana‐zirconia‐supported nickel catalysts and are abbreviated as 5NixSr/LaZr (x=1–4 wt. % Sr) and 5Ni/LaZr.

### Catalyst Characterization

The catalyst samples’ surface parameters, including surface area, pore volume, and pore diameter, were analyzed using Micromeritics Tristar II 3020 with the help of dewar and liquid nitrogen. The surface area and pore size analysis were performed using the Brunauer–Emmett–Teller (BET) equation and Barrett–Joyner–Halenda (BJH) pore size distribution, respectively. X‐ray powder diffraction (XRD) study was undertaken using a Rigaku Ultima 4 diffractometer and Cu Kα radiation (wavelength 1.54056 Å) radiation source. The instrument was operated at 40 kV and 40 mA. The scanning range was 5–100. The phases were documented using standard JCPDS cards after analysis. Fourier transform infrared (FTIR) spectroscopy was recorded employing IR Prestige‐21 SHMADZU. Raman analysis of fresh and spent catalyst samples was performed using a Laser Raman (NMR‐4500) Spectrometer (JASCO, Japan) over a 150–1250 cm^−1^ range. The 532 nm wavelength beam was used for excitation. Laser intensity was adjusted to 1.6 mW for 10 seconds of exposure time with 3 accumulations. The Spectra Manager Ver.2 software (JASCO, Japan) was used to process the spectra. H_2_‐temperature programmed reduction (TPR), CO_2_‐temperature programmed desorption (TPD), O_2_‐temperature programmed oxidation (TPO), and CO_2_‐temperature programmed oxidation (TPO) studies were conducted using Micromeritics Auto Chem II 2920 (USA). 70 mg of the sample was heated up to 900 °C at a heating rate of 10 °C/min under 40 ml/min flow rate of 10 % H_2_‐Ar for H_2_‐TPR, 10 % O_2_/He mixture for O_2_‐TPO and 10 % CO_2_/He mixture for CO_2_‐TPO. For CO_2_‐TPD, 70 mg of the sample was cleaned using helium flow at 200 °C for 1 hour. Then, it was fed with a mixture of 10 % CO_2_ and helium at a 30 ml/min flow rate at 50 °C for 30 minutes. CO_2_ was desorbed with a linear temperature increase up to 800 °C. The change in conductivity due to the consumption and desorption of gases over the catalyst surface was recorded by a temperature conductivity detector (TCD).

### Catalyst Activity Test

0.1 g of NixSr/LaZr (where x=1–4 wt.% Sr) catalyst is packed in a 9.1 mm diameter and 300 mm height tubular stainless‐steel reactor (PID Eng. and Tech Microactivity Reference Company). A K‐type thermocouple is inserted axially into the catalyst bed to monitor its temperature. To prepare the catalyst for DRM, a reductive pre‐treatment is performed under a 30 ml/min H_2_ flow for 1 hour at 700 °C. The reactor was flushed with N_2_ for 15 minutes to remove any physiosorbed H_2_. The dry reforming of methane (DRM) reaction was carried out at 700 °C by passing CO_2_, CH_4_, and N_2_ at a 30 : 30 : 10 ml/min molar ratio. This generated 42,000 mL/h⋅gcat of gas hourly space velocity (GHSV). The effluent is examined by an online GC equipped with molecular sieve 5 A and Porapak Q columns and a TCD detector. H_2_ and CO conversions were estimated by the following expressions:

(1) $$H_{2}\;Yield\;\left(\%\right)=\;\frac{Mole\;of\;H_{2}\;in\;Product}{2\;X\;{Mol\;of\;CH}_{4,\;in}}\;\times 100$$

(2)

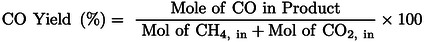




## Results and Discussion

### Characterization Results

**Figure 1:**
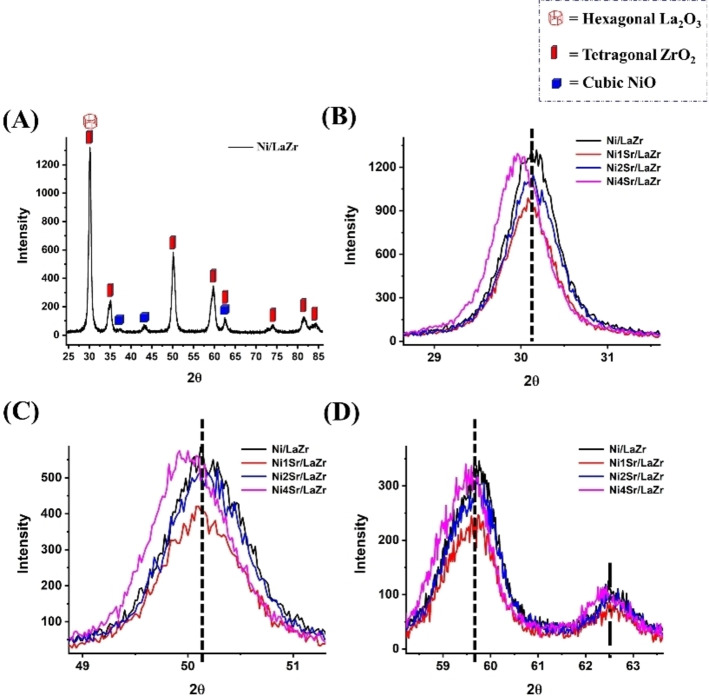
X‐ray diffraction pattern of 5Ni/LaZr and 5NixSr/LaZr (x=1–4 wt.%) catalysts.

X‐ray diffraction pattern of 5Ni/LaZr and 5NaxSr/LaZr (x=1–4 wt.%) catalysts are shown in Figure 1. Lanthana‐zirconia supported catalyst has tetragonal zirconia phase (at Bragg's angle 2θ=30.2°, 35.2°, 50.08°, 59.79°, 62.46°, 74.0°, 81.6° and 84.4°; JCPDS reference number 01–079‐1769), hexagonal lanthana phase (at Bragg's angle 2θ=30.2°; JCPDS reference number 01–074‐1144) and cubic NiO phase (at Bragg's angle 2θ=37.39°, 43.33°, 62.46°; JCPDS reference number 01–073‐1519). A stable tetragonal zirconia phase is formed. In literature, mixed oxide formation by “SrO and ZrO_2_” and “La_2_O_3_ and ZrO_2_ were reported.^[16]^ But here, no mixed oxide phases exist over the catalyst. However, the addition of strontium or La_2_O_3_ may induce the crystallinity of tetragonal ZrO_2_. Previously, over lead zirconate titanate, the addition of SrO was found to decrease the particle size, whereas particle size was increased by the addition of La_2_O_3_.^[17]^ Here, upon addition of 1 wt.% strontium over lantana‐zirconia supported Ni catalyst, the peaks intensity about 30.2°, 50.1°, 59.8° and 62.5° (related to tetragonal zirconia phase and lanthana phase) are decreased due to smaller grain size.^[18]^ However, a further increase in Sr loading causes a rise in peak intensity. Interestingly, upon 4 wt.% strontium loading, the peak intensity in diffraction patterns is not only increased but also shifted to lower Bragg's angle. It indicates that after an optimum strontium loading, the catalyst's lattice parameters are expanded.^[19]^

**Figure 2:**
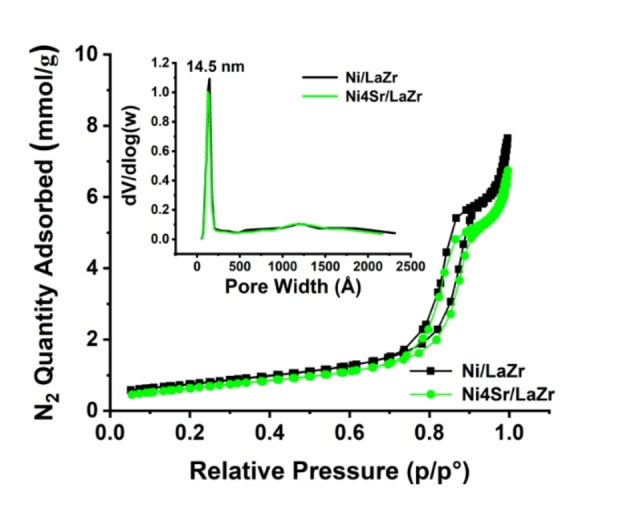
The adsorption isotherm of Ni/LaZr and Ni4Sr/LaZr catalysts.

The adsorption‐desorption plot and porosity distribution of 5Ni/LaZr and 5Ni4Sr/LaZr catalyst are shown in Figure 2. The data for surface area, pore volume, and pore diameter of 5Ni/LaZr and 5NixSr/LaZr (x=1–4 wt %) catalyst are shown in Table 1. The Catalyst shows types IV adsorption isotherm with H1 hysteresis loop, confirming the presence of mesoporous domain. The pores size distribution of catalysts is monomodal in the 14.5 nm range (inset of Figure 2). The average pore size of 5Ni/LaZr and 5NixSr/LaZr (x=1–4 wt.%) catalysts are also found in the 14.6–14.2 nm range. Up to 2 wt.% loading of strontium over 5Ni/LaZr, the surface area and pore volume drop marginally (Table 1). Upon higher loading of Sr (3–4 wt.%), the surface area is decreased substantially.

**Table 1:** Surface area, pore volume, and pore diameter of Ni/LaZr and NixSr/LaZr (x=1–4 wt.%) catalysts.

Catalyst	Surface area (m^2^/g)	Pore Volume (cm^3^/g)	Pore Diameter (nm)
Ni/LaZr	60.8	0.28	14.6
Ni1Sr/LaZr	60.8	0.29	14.7
Ni2Sr/LaZr	59.9	0.28	14.6
Ni3Sr/LaZr	57.3	0.26	14.1
Ni4Sr/LaZr	53.4	0.25	14.2

**Figure 3:**
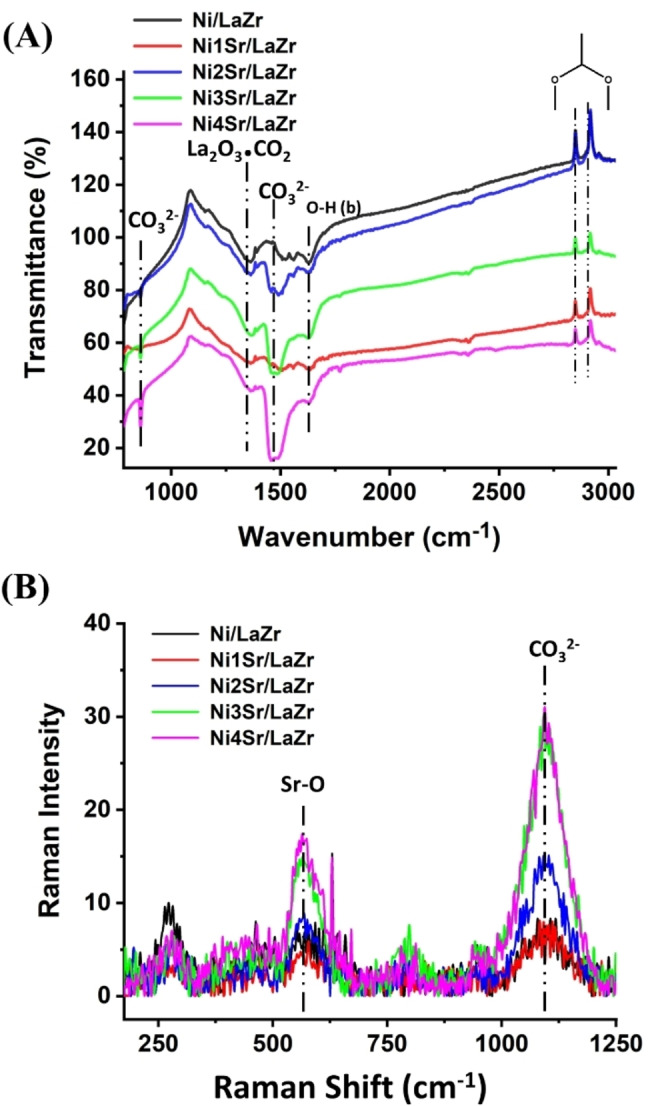
(A) Infra‐red spectra of Ni/LaZr and 5NixSr/LaZr (x=1–4 wt %) catalysts (B) Raman spectra of Ni/LaZr and NixSr/LaZr (x=1–4 wt %) catalysts.

Fourier transforms infrared spectra and Raman spectra of 5Ni/LaZr and 5NixSr/LaZr (x=1–4 wt.%) catalysts are shown in Figure 3. FTIR spectra of the Ni/LaZr catalyst sample show the vibration peaks for bi‐coordinated formate species at 2850 cm^−1^ and 2925 cm^−1^ and oxycarbonate species strongly coordinated to La^3+^ at 1370 cm^−1[20]^ (Figure 3A). Interestingly, upon promotional addition of strontium, the vibrational spectra for ionic carbonate species at 860 cm^−1^ and 1464 cm^−1^ appear.^[15]^ The vibration peaks for ionic carbonate species are intensified upon increasing the wt % of Sr in the Ni/LaZr catalyst. It can be said that ionic strontium carbonate is exclusively formed upon increasing loading of Sr over Ni/LaZr catalyst. On increasing loading of strontium over Ni/LaZr, the Raman vibration band at 560 cm^−1^ for Sr−O and 1071 cm^−1^ for CO_3_^2−^ are intensified[^21^, ^22^] (Figure 3b). The presence of strontium carbonate is once again detected on a Ni/LaZr catalyst that has been promoted with strontium.

**Figure 4:**
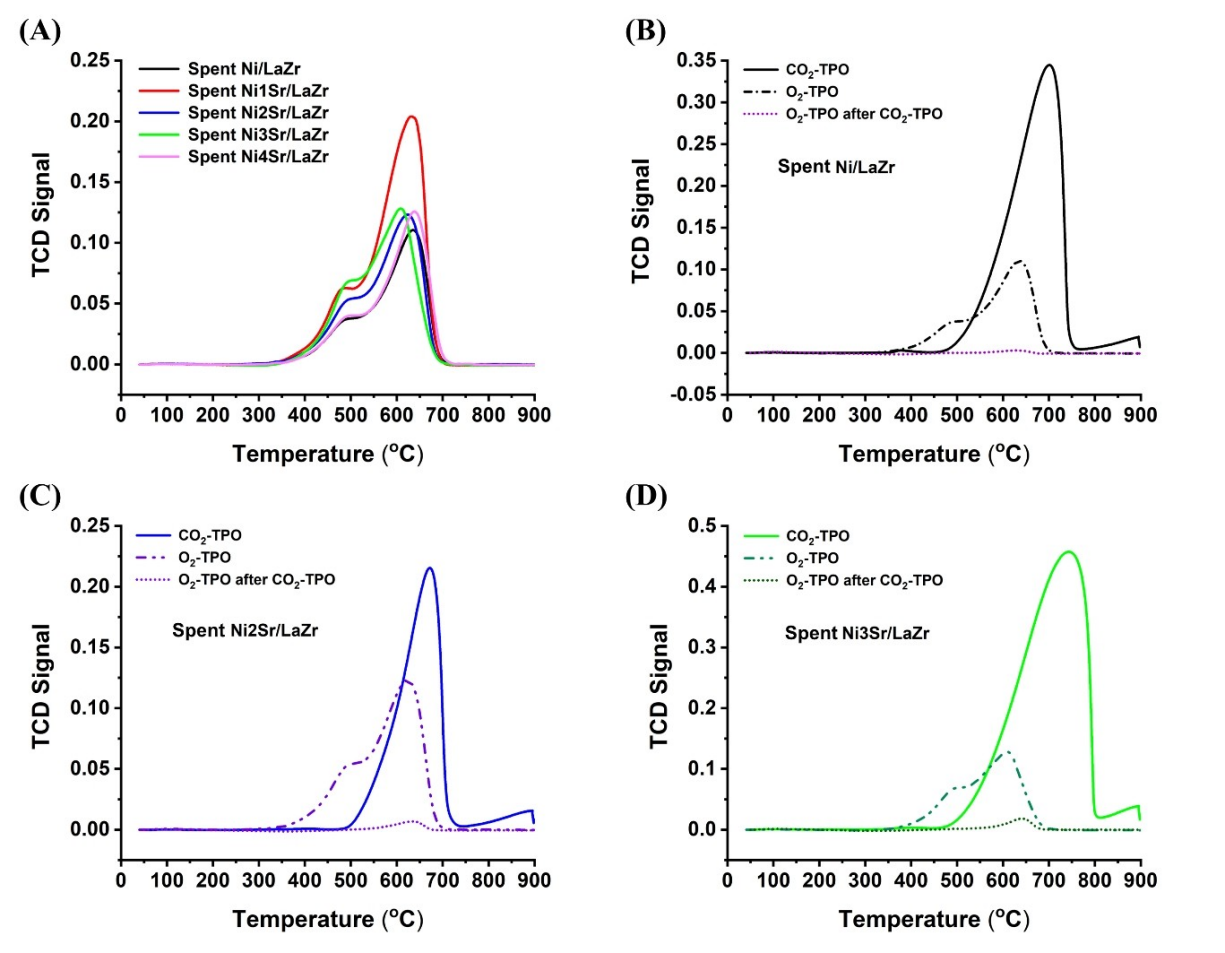
(A) O_2_‐TPO of spent‐Ni/LaZr and spent‐NixSr/LaZr (x=1–4 wt.%) catalysts (B) CO_2_‐TPO, O_2_‐TPO and “O_2_‐TPO after CO_2_‐TPO” of Ni/LaZr (C) CO_2_‐TPO, O_2_‐TPO and “O_2_‐TPO after CO_2_‐TPO” of Ni2Sr/LaZr (D) CO_2_‐TPO, O_2_‐TPO and “O_2_‐TPO after CO_2_‐TPO” of Ni4Sr/LaZr.

To understand the oxidizing capacity of carbon deposits over spent catalyst systems under oxidizing gas like CO_2_ and O_2_, temperature‐programmed oxidation studies are carried out and shown in Figure 4. The O_2_‐TPO peak is observed at about 475 °C and >600 °C. The earlier one is attributed to amorphous carbon (sp^2^ C‐atoms), whereas the latter one is to inert carbon (sp^3^ C‐atoms).^[23]^ (Figure 4A). Upon incorporation of 1 wt.% Sr to Ni/LaZr catalyst, a relatively higher intensity peak is observed in O_2_‐TPO which indicates the presence of more amount of coke over Ni1Sr/LaZr than unpromoted catalyst. Upon increasing the strontium loading to 2 wt %, the peak intensity of O_2_‐TPO is decreased. Previously, infrared spectra confirmed the presence of a higher amount of CO_2_‐surface interacting species upon 2 wt % Sr loading over Ni/LaZr which carries out pronounce oxidation of carbon and results in less coke deposit over the catalyst surface. A single intense peak of about 700 °C is observed under CO_2_‐TPO for the same catalyst. Oxidation of carbon deposit by CO_2_ at this temperature is markable because the reaction temperature over the current catalyst system is also set at 700 °C. For every catalyst, the intensity of the CO_2_‐TPO profile of each catalyst is higher than the O_2_‐TPO profile of the same catalyst (Figure 4B–D). It indicates that CO_2_ has more oxidizing power for carbon deposit oxidation than O_2_. Further, O_2_‐TPO is carried out just after CO_2_‐TPO, and the oxidation profile of the catalyst is worst (Figure 4B–D). It indicates that most of the oxidizable carbon deposit is oxidized by CO_2,_ and very few carbon deposits are left behind for oxidation with O_2_. It is clear that CO_2_ is a promising oxidizing gas in DRM, and its oxidation potential is higher than O_2_ over the current catalyst system.

**Figure 5:**
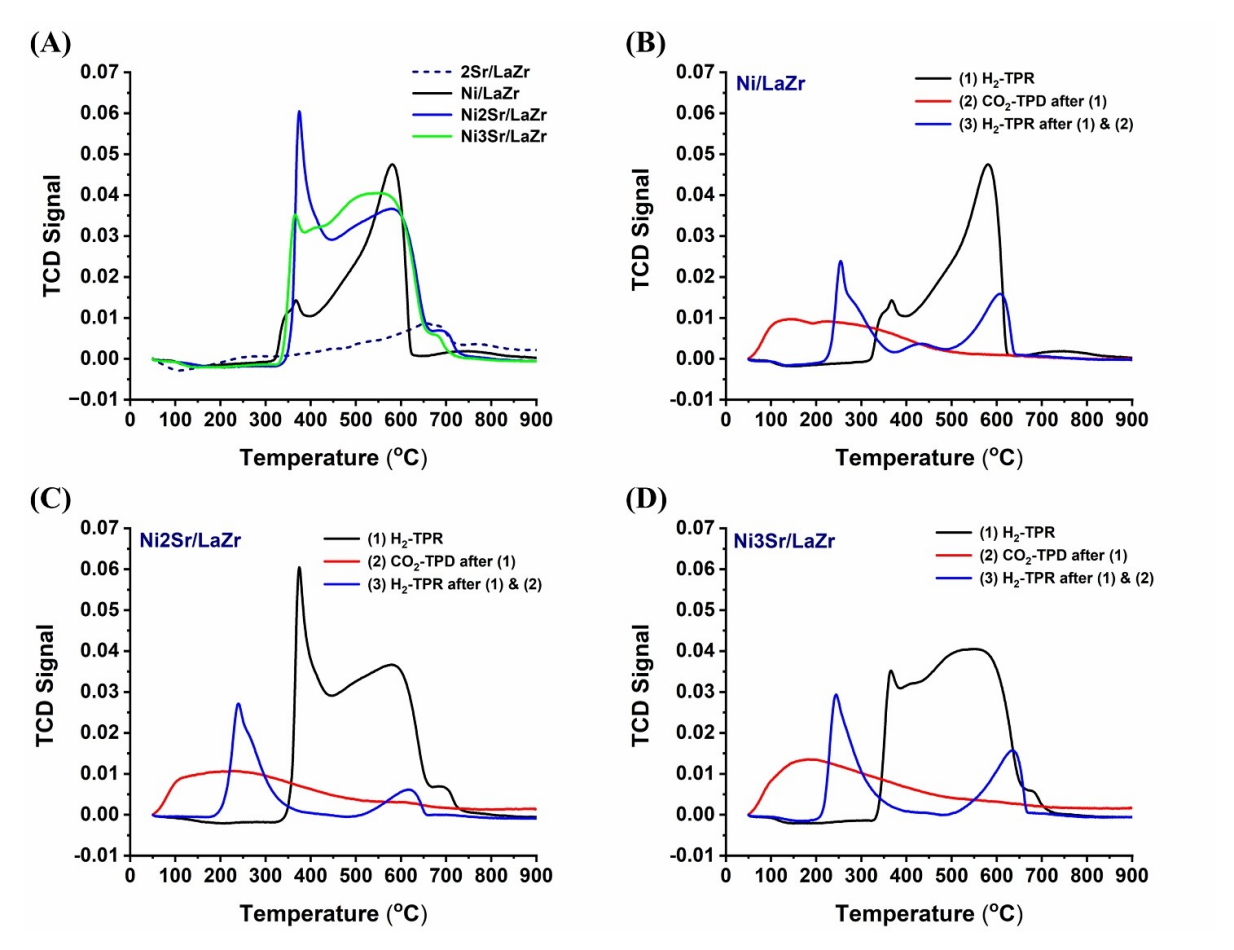
(A) H_2_‐TPR of 2Sr/LaZr, Ni/LaZr and NixSr/LaZr (x=1–3 wt.%) catalysts (B) Cyclic H_2_TPR‐ CO_2_‐TPD‐H_2_TPR profile of Ni/LaZr catalyst (C) Cyclic H_2_TPR‐ CO_2_‐TPD‐H_2_TPR profile of Ni2Sr/LaZr catalyst (D) Cyclic H_2_TPR‐ CO_2_‐TPD‐H_2_TPR profile of Ni3Sr/LaZr catalyst.

The current catalyst system is reduced under hydrogen before the dry reforming of methane. The sole target of this reductive pretreatment is to prepare the catalytic active metallic Ni from NiO. To estimate the population of such reducible NiO species, H_2_‐Temperature programmed reduction of the catalyst is carried out (Figure 5). In previous publication, H_2_‐TPR of Lanthana‐Zirconia sample showed no consumption of H_2_.^[24]^ Upon addition of Sr over LaZr, a small reduction peak about 700–750 °C is observed (Figure 5A). It clearly indicates that this reduction peak is due to reduction of Sr‐related compounds. In the literature, the reduction peak in this temperature range was reported for reduction of SrCO_3_ species into SrO.[^5^, ^25^] It can be expected that during calcination, CO_2_ (from environment) reacts with SrO and forms SrCO_3_.^[6]^ So here, the reduction peak about 700–750 °C is claimed to reduction of SrCO_3_ into SrO under H_2_ atmosphere. The reductive profile of rest catalysts can be divided into two regions: a reductive peak before 400 °C (peak maxima about 350 °C) and a broad peak at 400–800 °C temperature. The low‐temperature reduction peak is for easily reducible NiO‐interacted species, whereas the high‐temperature reduction peak is for less readily (moderately) reducible NiO‐interacted species. Interestingly, the Ni/LaZr catalyst has a low amount of easily reducible NiO‐interacted species and a high population of moderately reducible NiO‐interacted species. Upon the addition of 2 wt % Sr over Ni/LaZr, the reduction profile in the low‐temperature region is intensified.[^26^, ^27^] It indicates the growth of easily reducible NiO‐interacting species upon addition of 2 wt % strontium. Upon 3 wt.% Sr addition, the population of easily reducible NiO‐interacted species are decreased markedly. Predictions were made about the distribution of different reducible NiO species in H_2_‐TPR. However, it is important to note that CO_2_ acts as an oxidizing gas during the dry reforming of methane. This means that it can oxidize the carbon deposit and metallic Ni (active site). If active sites are oxidized, the catalyst will lose the active sites for reaction. Again, H_2_ gas is formed as a product during DRM, which is again a reducing gas and can reduce the NiO further. Overall, it can be said that under the oxidizing (CO_2_) and reducing gas stream (H_2_) during the DRM reaction, the distribution of active sites is further modified, which regulates the catalytic activity in the long run. To understand the population of reducible NiO‐species under the oxidizing and reducing atmosphere, we have carried out cyclic H_2_‐TPR, CO_2_‐TPD, and H_2_‐TPR in continuation in the target of reducing NiO into metallic Ni by H_2_, then oxidizing metallic Ni into NiO by CO_2_ and finally reducing the NiO into metallic Ni respectively (Figure 5B–D). It is observed that the reducibility pattern is shifted to a lower temperature. It indicates the increasing reducibility of the catalyst under the oxidising and reducing gas stream during the DRM.

**Figure 6:**
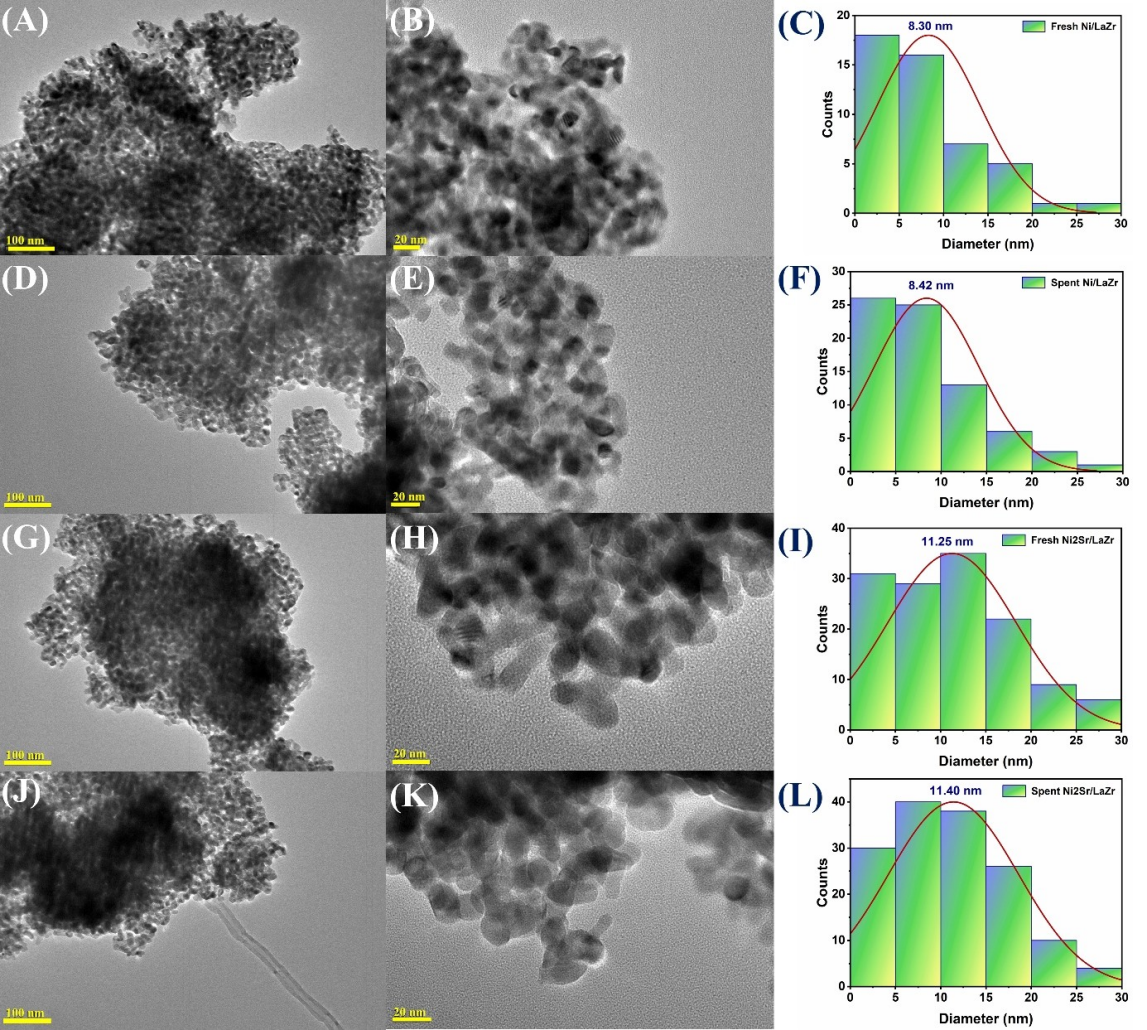
TEM image of fresh Ni/LaZr at different scales (A) 100 nm (B) 20 nm (C) Particle size distribution graph of Fresh Ni/LaZr. TEM image of spent Ni/LaZr at different scales (D) 100 nm (E) 20 nm (F) Particle size distribution graph of Spent Ni/LaZr. TEM image of fresh Ni2Sr/LaZr at different scales (G) 100 nm (H) 20 nm (I) Particle size distribution graph of Fresh Ni2Sr/LaZr. TEM image of spent Ni2Sr/LaZr at different scales (J) 100 nm (K) 20 nm (L) Particle size distribution graph of Spent Ni2Sr/LaZr.

The Transmission electron microscopy (TEM) image of (Ni/LaZr) and 2 wt.% Sr promoted Ni/LaZr catalysts are shown in Figure 6. The particle size of NiO over nonpromoted (Ni/LaZr) and 2 wt.% Sr promoted Ni/LaZr catalysts are found at 8.30 nm and 11.25 nm respectively. After the DRM reaction, the particle size of NiO over Ni/LaZr and Ni2Sr/LaZr catalysts are grown to 8.42 nm and 11.40 nm respectively. It indicates the nominal growth of NiO during the DRM reaction.

### Catalyst Activity Results

**Figure 7:**
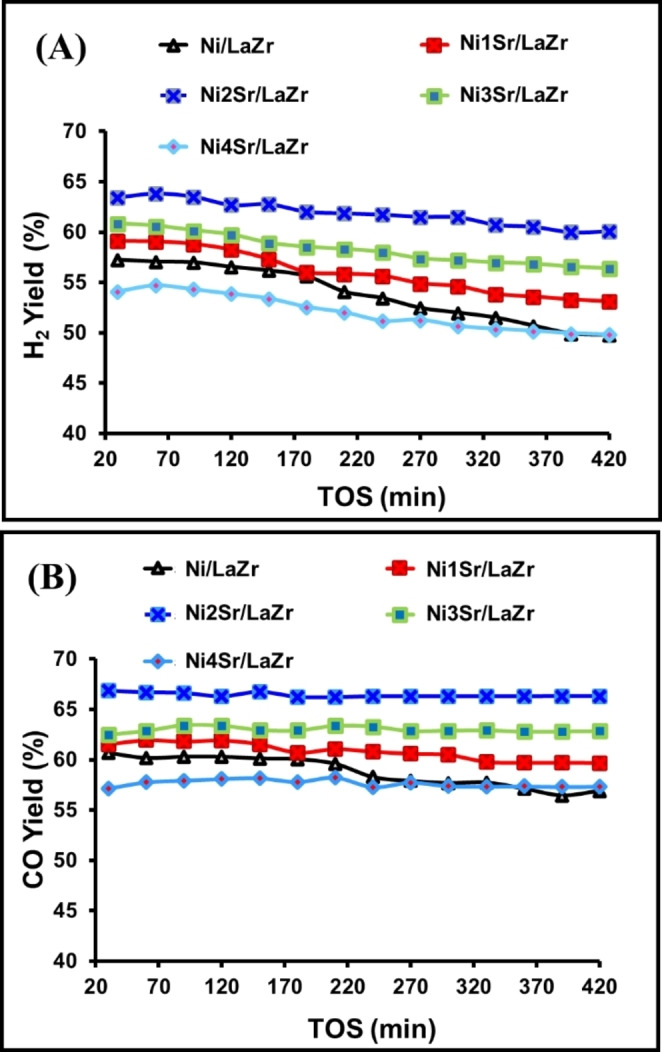
Catalytic activity results: (A) H_2_ yield (%) vs time on stream study of Ni/LaZr and NixSr/LaZr (x=1–4 wt.%) catalysts (B) CO yield (%) vs time on stream study of Ni/LaZr and NixSr/LaZr (x=1–4 wt.%) catalysts.

The H_2_ yield (%) vs time on stream, CO yield (%) vs time on stream, and “H_2_ yield (%) and CO yield (%)” vs time on stream studies of Ni/LaZr and NixSr/LaZr (x=1–4 wt.%) catalysts are shown in Figures 7 and S1. The phase transition of monoclinic ZrO_2_ phases is a major limitation of Ni/ZrO_2_ towards high‐temperature catalytic application.^[28]^ But here, upon the addition of 9 wt.% La_2_O_3_ to 91 wt % ZrO_2_, the tetragonal phase of ZrO_2_ is nurtured over Ni/LaZr catalyst, which is thermally stable and suitable for high‐temperature DRM reaction. Ni/LaZr catalyst shows an initial 57 % H_2_ yield and 61 % CO yield, which slows down to 50 % H_2_ yield and 57 % CO yield with 420 minutes time on stream (TOS). The H_2_ yield and Co yield are progressed to 59–53 % and 62–60 % (during 420 minutes TOS), respectively, upon the addition of 1 wt.% Sr over Ni/LaZr catalyst. 2 wt.% Sr loading over Ni/LaZr results in the highest 64–60 % H_2_ and ~67 % CO yield during 420 minutes TOS. When more than 2 wt % of strontium is loaded onto Ni/LaZr, the catalyst's performance toward DRM decreases.

## Discussion

Some common observations over the Ni/LaZr and NixSr/LaZr (x=1–4 wt.%) catalysts system should be briefed. Over the Ni/LaZr and NixSr/LaZr (x=1–4 wt.%) catalysts, CO_2_ is found to be a better oxidizing gas for oxidizing carbon deposits than O_2_ (confirmed by CO_2_‐TPO, O_2_‐TPO, and O_2_‐TPO after CO_2_‐TPO results). The second observation is that during the DRM reaction, oxidising and reducing gases are present over the catalyst, which further modifies the distribution of active sites and induces a greater edge of reducing (confirmed by cyclic H_2_‐TPR‐CO_2_‐TPD‐ H_2_‐TPR experiments).

**Figure 8:**
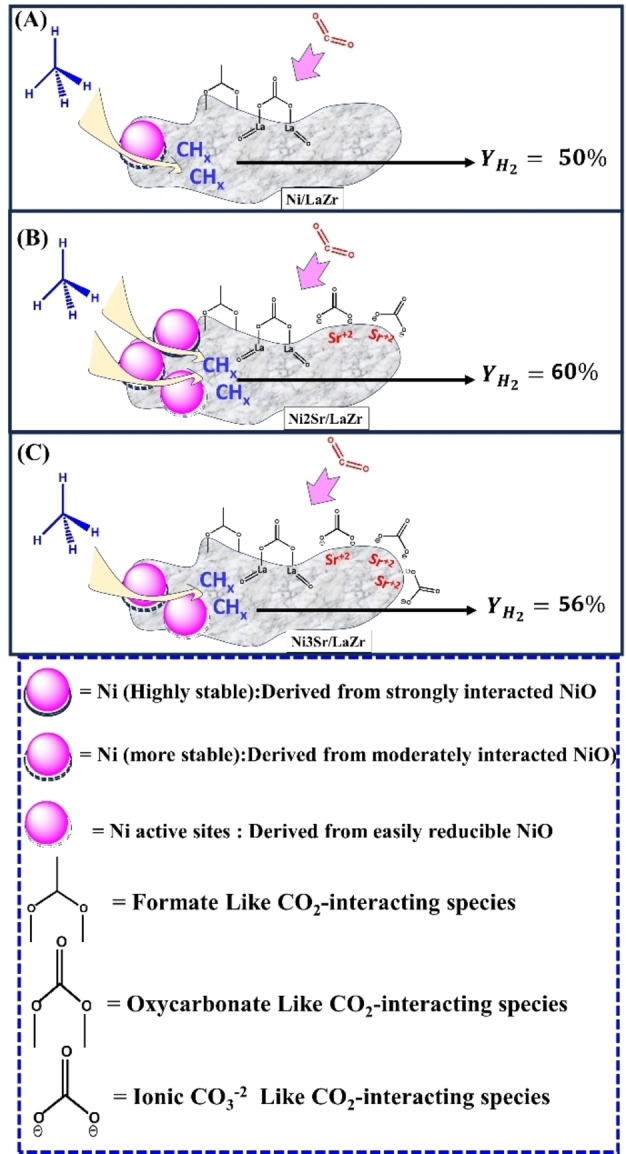
The proposed reaction pathways over (A) Ni/LaZr catalyst, (B) Ni2Sr/LaZr catalyst, and (C) Ni3Sr/LaZr catalyst.

Based on characterization and activity results over Ni/LaZr and NixSr/LaZr (x=1–4 wt.%) catalysts, the possible reaction pathways are presented in Figure 8. The Ni/LaZr catalyst has a high concentration of active sites (Ni) derived from moderately reducible NiO‐interacted species. CO_2_ interacts with the catalyst surface and forms formate and lanthanum oxycarbonate even under normal environmental conditions (confirmed by infrared spectroscopy). During the DRM reaction, the oxidizing gas stream (CO_2_) and reducing gas stream (H_2_) exist, which induces a higher edge of reducibility further (confirmed by cyclic H_2_TPR‐CO_2_TPD‐H_2_TPR experiment). The CO_2_‐TPO experiment shows excellent oxidation capacity of carbon deposit about reaction temperature 700 °C. Overall, it can be said that the active sites population over Ni/LaZr carry out CH_4_ decomposition, the decomposed‐CH_4_ (CH_4−x_; x=1,2,3,4) is oxidized by adsorbed‐CO_2_ species (like formate and lanthanum oxycarbonate) and gives 57 % H_2_ yield and 61 % CO yield. After 420 minutes, 50 % H_2_ yield and 57 % CO yield are retained over Ni/LaZr catalyst. The higher CO yield than H_2_ yield indicates that H_2_‐consuming reaction like reverse water gas shift reaction is also exist over the catalyst.

Interestingly, upon loading of 1 wt.% Sr over Ni/LaZr, ionic carbonate species are also grown. The pronounced oxidation of carbon deposits due to the presence of formate, oxycarbonate, and ionic CO_3_^2−^ can be expected over the Ni1Sr/LaZr catalyst. It results in 59–53 % H_2_ yield and 62–60 % CO yield during the 420‐minute time on stream over Ni1Sr/LaZr catalyst. Interestingly, O_2_‐TPO results shows the presence of more amount of coke Ni1Sr/LaZr than unpromoted catalyst. However, the additional coke formation does not affect the catalytic activity over Ni1Sr/LaZr. It indicates that the active sites remain exposed even after deposition of higher amount of carbon. It was reported that if rate of coke formation (at active sites) is firmly matched with the rate of coke diffusion, the active sites of catalyst remains exposed and the catalytic activity toward DRM was not affected.[^21^, ^29^] Here also, the catalytic activity is not affected even after higher coke deposition due to timed diffusion of coke (away from the active sites) over Ni1Sr/LaZr. Upon further loading of Sr (2 wt.%), both the reducibility of NiO and the population of ionic CO_3_^2−^ species are increased (confirmed by infrared and Raman spectroscopy). Ni2Sr/LaZr catalyst has a wide range of active sites, “Ni,” which is derived from NiO at temperatures as well as various CO_2_‐surface interacting species which triggers the best catalytic activity toward DRM. 64–60 % H_2_ yield and Ni2Sr/LaZr catalyst and ~67 % CO yield is achieved over Ni2Sr/LaZr catalyst during 420‐minute time on stream. It seems that after further loading of Sr, the population of active sites is not increased but the population of ionic CO_2_‐species is increased, and it results in relatively inferior activity. Ni3Sr/LaZr catalyst shows 61–56 % H_2_ yield and 63 % CO yield during 420 minutes. It indicates that ionic CO_3_^2−^ species concentrations greater than an optimum level may harm the catalytic activity. Possibly at higher CO_3_^2−^ concentrations, stable carbonates may form which may not be dissociated during the oxidation of the carbon deposit.^[30]^ In the same line, the intensity of CO_3_^2−^ species is observed maximum over Ni4Sr/LaZr but the catalytic activity over Ni4Sr/LaZr is found even less than unpromoted catalyst.

The comparative study of catalytic activity in terms of H_2_ yield over different catalyst systems are shown in Table 2. In mean of silica, alumina and alumina‐silica based Ni supported catalysts, the maximum H_2_ yield are achieved over Ni/SiO_2_ (prepared by oleic acid assisted impregnation method), 5Ni/Pd+Al_2_O_3_ catalyst and 5Ni2Ce/ZSM‐5 are found 48 % (during 360‐minute TOS), ~56 % (during 420‐minute TOS) and 43 % (during 300‐minute TOS) respectively.[^31^, ^32^, ^33^, ^34^, ^35^] Among stabilized‐zirconia based Ni supported catalysts; 5Ni/15YZr and 5Ni/WO_3_+ZrO_2_ and Ni/LaZr catalyst showed 55 % (during 420‐minute TOS), 49 % (during 470‐minute TOS) and 50 % H_2_ yield respectively.[^14^, ^35^, ^36^, ^37^, ^38^, ^39^, ^40^, ^41^, ^42^, ^43^] Upon promotional addition of 2 wt % Sr over 5Ni/WO_3_+ZrO_2,_ H_2_ yield was increased to 57 %. Over the current catalyst system also, upon addition of 2 wt % strontium over Ni/LaZr catalyst, H_2_ yield jumps to 60 % (against 50 % H_2_ yield over 5Ni/LaZr catalyst). This performance of 5Ni2Sr/LaZr is found best among the rest catalysts.

**Table 2:** The comparative study of catalytic activity in terms of H_2_ yield over different catalyst systems.

Sr no.	Catalyst name	Method	Weight of active metal (wt.%)	CW (%) in mg	I.D. (mm)	RT (°C)	Flow rate (cm^3^/min)			GHSV (cm^3^/h g_cat_) in mL	TOS (Min)	$Y_{H_{2}}$ (%)	Refs.
							CH_4_	CO_2_	Inert Gas				
							(in mL)/min						
1	Ni‐SP‐Imp	WI	0.2 (Ni)	100	6	700	20	20	0	24000	360	37	[31]
2	Ni‐SP‐OA	WI	0.2 (Ni)	100	6	700	20	20	0	24000	360	48	[31]
3	Ni/Pd‐SP‐Imp	WI	0.2 (Ni)**+**0.2 (Pd)	100	6	700	20	20	0	26000	360	41	[31]
4	5Ni/Pd+Al_2_O_3_	WI	5 (Ni)	100	9.1	800	30	30	10	42000	420	55.8	[32]
5	5Ni3TiAl	WI	5 (Ni)	100	9	700	30	30	10	42000	420	30	[33]
6	5Ni3MoAl	WI	5 (Ni)	100	9	700	30	30	10	42000	420	39	[33]
7	5Ni/Al_2_O_3_‐SiO_2_	WI	5 (Ni)	100	7	700	15	15	0	18000	300	40	[34]
8	5Ni2Ce/ZSM‐5	WI	5 (Ni)	150	9.1	700	30	30	10	42000	300	43	[35]
9	5Ni2CS/ZSM‐5	WI	5 (Ni)	150	9.1	700	30	30	10	42000	300	23	[35]
10	5Ni2Sr/ZSM‐5	WI	5 (Ni)	150	9.1	700	30	30	10	42000	300	28	[35]
11	5Ni2Cu/ZSM‐5	WI	5 (Ni)	150	9.1	700	30	30	10	42000	300	3.4	[35]
12	5Ni2Ce/ZSM‐5	WI	5 (Ni)	150	9.1	700	30	30	10	42000	300	10.6	[35]
13	Ni/CeZr	WI	5 (Ni)	–	0.53	750	30	20	50	120000	1680	33	[36]
14	5Ni1Ce/Zr	WI	5 (Ni)	100	9.1	700	30	30	10	42000	420	47	[37]
15	Ni/CZ28	WI & CP(Support)	5 (Ni)	100	10	700	50	50	0	30000	180	34	[38]
16	5Ni/Zr	WI	5 (Ni)	100	9.1	700	30	30	10	42000	420	43	[37]
17	5Ni/15YZr	WI	5 (Ni)	100	9.1	700	30	30	10	42000	420	55	[14]
18	5Ni/5MgZr	WI	5 (Ni)	100	9.1	700	30	30	10	42000	420	23	[14]
19	5Ni/MgO+ZrO_2_	WI	5 (Ni)	100	9.1	700	30	30	10	42000	420	46	[39]
20	5Ni/WZr	WI	5 (Ni)	100	9	700	30	30	10	42000	420	45	[40]
21	5Ni/WO_3_+ZrO_2_	WI	5 (Ni)	100	9.1	700	30	30	10	42000	470	49	[41]
22	5Ni2Sr/WO_3_+ZrO_2_	WI	5 (Ni)	100	9.1	700	30	30	10	42000	470	57	[41]
23	5Ni2Sr/WO_3_+ZrO_2_	WI	5 (Ni)	100	9.1	700	30	30	10	42000	1800	50	[41]
24	Ni/LaZr‐ns	UHL	5 (Ni)	100	4	400	10	10	80^#^	7200	6000	4.8	[42]
25	Ni/LaZr‐Macropores	UHL	5 (Ni)	100	4	400	10	10	80^#^	7200	6000	5.6	[42]
26	Ni/LaZr‐Mesopores	UHL	5 (Ni)	100	4	400	10	10	80^#^	7200	6000	3.1	[42]
27	Ni/LaZr‐Mesopores	UHL	5 (Ni)	100	4	400	10	10	80^#^	14400	10800	4.8	[42]
28	Ni/LaZr	WI	5 (Ni)	–	0.53	750	30	20	50	120000	1680	30	[36]
29	La_1.95_Sr_0.05_Zr_1.904_Ni_0.096_O_7‐d_	PM	–	75	6	900	10	10	0	24000	2700	27.5	[43]
30	La_1.95_Sr_0.05_Zr_1.44_Ni_0.56_O_7‐d_	PM	–	75	6	900	10	10	0	24000	2700	54.6	[43]
31	Ni/LaZr	WI	5 (Ni)	100	9.1	700	30	30	10	42000	420	50	This Study
32	Ni2Sr/LaZr	WI	5 (Ni)	100	9.1	700	30	30	10	42000	420	60.11	

CW: Catalysts Weight, I.D.: Initial Diameter of fixed bed tubular flow reactor, Method: WI: Wet impregnation method, CP: Co‐Precipitation method, UHL: Urea Hydrolysis Method, PM: Pechini Method, RT (°C): Reaction Temperature, CT (°C): Calcination Temperature, GHSV: Gas Hourly Space Velocity, TOS: Time on Stream, Y_H2_ (%): Hydrogen Yield (%), SP‐OA : SP: Mesoporous SiO_2_ (316 m^2^/g, pre diameter=11.81 nm), OA: Oleic acid assisted, #: Sign dedicated to ether.

## Conclusions

The Ni/LaZr and Ni2Sr/LaZr catalyst system is thermally stable; it surges active sites (Ni) upon reduction under H_2_ (during reductive pretreatment) and has an adequate population of interacted CO_2_ species. The active sites carry out CH_4_ decomposition, whereas CO_2_ oxidizes decomposed‐CH_4_ or CH_4−x_ (x=1–4) in DRM. It is markable that all catalysts get a higher edge of reducibility further under oxidizing and reducing gas during the DRM reaction. The 50 % H_2_ yield is achieved over Ni/LaZr catalyst, which is facilitated with Ni active sites derived from moderately interacted NiO‐species as well as format and oxycarbonate‐like CO_2_‐interacted species. It has been observed that the highest activity over Ni2Sr/LaZr occurs due to the presence of a variety of interacted Ni active sites. These sites are derived from easily reducible NiO, moderately interacted NiO, and strongly interacted NiO. Additionally, the presence of ionic CO_3_^2−^ species, along with other CO_2_‐interacted species, contributes to the high activity. However, the excessive presence of ionic carbonate upon higher Sr loading (>2 wt.%) has been found to decrease the catalytic activity.

## Supporting Information Summary

Figure S1 “H_2_ yield (%) and CO yield (%)” vs time on stream study of Ni/LaZr and NixSr/LaZr (x=1–4 wt.%) catalysts.

## Conflict of Interests

The authors declare no conflict of interest.

1

## Supporting information

As a service to our authors and readers, this journal provides supporting information supplied by the authors. Such materials are peer reviewed and may be re‐organized for online delivery, but are not copy‐edited or typeset. Technical support issues arising from supporting information (other than missing files) should be addressed to the authors.

Supporting Information

## Data Availability

No data was used for the research described in the article.
