# Supplementary material for: Role of Active Site and CO2‐Interacting Surface Species in Dry Reforming of Methane over Strontium Promoted Ni Catalyst Supported by Lanthanum‐Zirconia
Source: ChemistryOpen. 2024 Nov 6;14(1):e202400151. doi: 10.1002/open.202400151 (PMC11726706; doi:10.1002/open.202400151)
Supplement: Supplementary file 1 — Supporting Information [file OPEN-14-e202400151-s001.pdf]

ChemistryOpen

Supporting Information

Role of Active Site and CO_2-Interacting Surface Species in Dry Reforming of Methane over Strontium Promoted Ni Catalyst Supported by Lanthanum-Zirconia

Kenit Acharya, Ahmed S. Al-Fatesh,* Anis H. Fakeeha, Ahmed E. Abasaeed, Othman Alothman, Hammad Ahmad Jan, Naif Alarifi, Jehad K. Abu-Dahrieh,* and Rawesh Kumar*

Role of Active Site and CO_2-interacting Surface Species in Dry Reforming of Methane over Strontium Promoted Ni Catalyst Supported by Lanthanum-zirconia

Kenit Acharya[a], Ahmed S. Al-Fatesh[b*], Anis H. Fakeeha[b], Ahmed E. Abasaeed[b], Othman Alothman[b], Hammad Ahmad Jan[c], Naif Alarifi[d], Jehad K. Abu-Dahrieh[e*], Rawesh Kumar[a*]

[a] Kenit Acharya, Dr. Rawesh Kumar
Department of Chemistry, Indus University, Ahmedabad, Gujarat 382115, India.

[b] Ahmed S. Al-Fatesh, Ahmed A. Ibrahim, Anis H. Fakeeha, Ahmed E. Abasaeed, Othman Alothman
Chemical Engineering Department, College of Engineering, King Saud University P.O. Box 800, Riyadh 11421, Kingdom of Saudi Arabia.

[c] Hammad Ahmad Jan
Department of Botany, University of Buner, Swari, Pakistan.

[d] Naif Alarifi
Institute of Refining and Petrochemicals Technologies, King Abdulaziz City for Science and Technology (KACST), P.O. Box 6086, Riyadh 11442, Kingdom of Saudi Arabia.

[e] Jehad K. Abu-Dahrieh
School of Chemistry and Chemical Engineering, Queen's University, Belfast, BT9 5AG, UK.

E-mail:, aalfatesh@ksu.edu.sa , kr.rawesh@gmail.com , j.abudahrieh@qub.ac.uk

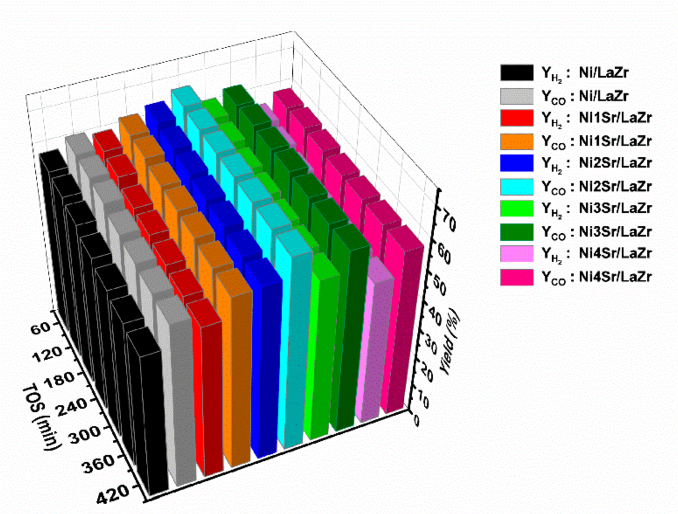

Figure S1. "H_2 yield (%) and CO yield (%)" vs time on stream study of Ni/LaZr and NixSr/LaZr (x =1-4 wt.%) catalysts.
